# Factors Affecting the Fetal Fraction in Noninvasive Prenatal Screening: A Review

**DOI:** 10.3389/fped.2022.812781

**Published:** 2022-01-27

**Authors:** Cechuan Deng, Shanling Liu

**Affiliations:** ^1^Prenatal Diagnostic Center, Department of Medical Genetics, West China Second University Hospital, Sichuan University, Chengdu, China; ^2^Key Laboratory of Birth Defects and Related Diseases of Women and Children, Ministry of Education, Sichuan University, Chengdu, China

**Keywords:** noninvasive prenatal screening, cell-free fetal DNA, fetal fraction, molecular genetics, genetic counseling

## Abstract

A paradigm shift in noninvasive prenatal screening has been made with the discovery of cell-free fetal DNA in maternal plasma. Noninvasive prenatal screening is primarily used to screen for fetal aneuploidies, and has been used globally. Fetal fraction, an important parameter in the analysis of noninvasive prenatal screening results, is the proportion of fetal cell-free DNA present in the total maternal plasma cell-free DNA. It combines biological factors and bioinformatics algorithms to interpret noninvasive prenatal screening results and is an integral part of quality control. Maternal and fetal factors may influence fetal fraction. To date, there is no broad consensus on the factors that affect fetal fraction. There are many different approaches to evaluate this parameter, each with its advantages and disadvantages. Different fetal fraction calculation methods may be used in different testing platforms or laboratories. This review includes numerous publications that focused on the understanding of the significance, influencing factors, and interpretation of fetal fraction to provide a deeper understanding of this parameter.

## Introduction

Circulating cell-free DNA has been proven to be useful for noninvasive oncological examinations and general medical examinations by numerous studies. Cell-free fetal DNA (cffDNA), originating from placental tissue and found in maternal plasma, was first reported in (1) and was initially used for fetal sex determination (2). With the advent of next-generation sequencing, two studies published in 2008 showed that cffDNA can be used to screen for common autosomal aneuploidies in fetuses (3, 4). Subsequently, cffDNA was included in trisomy 21 screening in (5). Various tests that use cffDNA to screen for fetal aneuploidies have been developed since then and are collectively called noninvasive prenatal screening (NIPS) (6).

**Figure 1:**
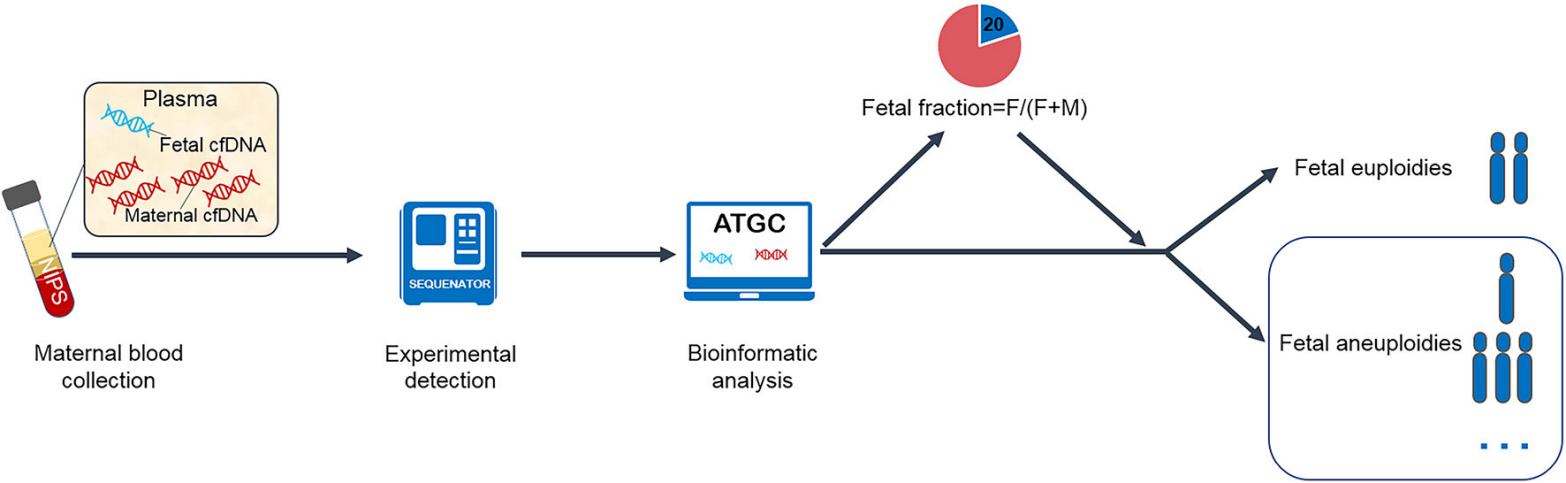
Flow chart of noninvasive prenatal screening.

The circulating cell-free DNA found in maternal plasma includes DNA of both maternal and fetal origins. Maternal circulating cell-free DNA originates from all maternal organs, including solid tumors, and mainly from the hematopoietic system. cffDNA is primarily derived from placental trophoblast cells and represents fetal DNA (placental DNA). Fetal fraction (FF) is the ratio of cffDNA to all circulating cell-free DNA in the maternal plasma (Figure 1). At 10–20 weeks of gestation (the most common time for NIPS), FF is ~10–15% (5, 7). During NIPS, maternal and fetal cell-free DNA is not separated, so it is imperative to understand FF to accurately interpret NIPS results. Substantial research on the FF has been performed to date, but it is relatively scattered. Therefore, an integrated review of FF is necessary. This review focuses on the significance and influencing factors of FF, and the management of pregnant women with failed NIPS results due to a low FF.

## Significance of FF

FF is an important parameter affecting the accuracy of NIPS for chromosomal aneuploidy. FF assessment at the time of NIPS is recommended as an essential part of quality control and statistical reliability determination to ensure that sufficient cffDNA is present in maternal plasma to obtain reliable results. Usually, a FF of 2–4% is set as the minimum threshold to obtain accurate NIPS results in the laboratories (8, 9).

Exceptionally high and low FF have different interpretations. A low FF may indicate a higher risk of aneuploidy, ranging from 2.7 to 23.3% (10–12). If a sample's FF is below the set threshold, it will be considered a “no-call” result, which typically has a rate of 2–5%. Therefore, setting a FF threshold requires a trade-off between maximizing the statistical reliability of NIPS and minimizing the failure rate. The prevalence of aneuploidy was significantly higher in pregnant women with a FF of <4% than in the entire cohort (4.7 vs. 0.4%) (11). Moreover, a sufficient FF of both trisomy 21 and trisomy 18 was required for NIPS to successfully identify the mosaic case (13). As long as FF is >4%, the FF of the group with false-positive and false-negative NIPS results was not lower than that of the group with true-positive results (14, 15).

However, a higher FF is not always better. An unusually increased FF may predict adverse pregnancy outcomes, such as spontaneous preterm birth (16). FF elevation in the first trimester (FF = 35.3%) was considered a possible marker of an abnormally invasive placenta (17). A FF of >40% was defined as a “no call” result (9). The usefulness of cffDNA and FF in predicting adverse pregnancy outcomes has not been adequately proven. No consensus on the maximum FF for achieving valid NIPS results is currently available.

Clinically speaking, a low trisomy score (Z-value or equivalent measure) relative to FF also indicated possible placental mosaicism (18). In addition, the mosaicism ratio was calculated as the ratio of the portion of cffDNA affected by aneuploidy to the total FF. A mosaicism ratio of <0.7 may indicate a mosaic fetal karyotype (19, 20).

All in all, FF within the normal range is an important link to ensure the accuracy of NIPS. Too low FF may affect the accuracy of NIPS and too high FF may indicate the presence of pregnancy complications. However, FF is not routinely reported in some laboratories. A systematic review published in 2018 included 30 studies, of which 6 examined FF in male fetuses only; 5 did not report or measure FF (21). The American College of Medical Genetics and Genomics (ACMG) recommended that all laboratories establish tests and efficacy analyses for FF and that FF be clearly identified in the NIPS report (22). Furthermore, it recommended that in the case of “no call” NIPS reports, the reason should be clarified (23). The purpose of FF report is to inform recipients that NIPS has detected sufficient cffDNA at the time of NIPS. Once FF is reported, the link between FF and NIPS results should be clarified.

## Factors Affecting FF

The factors that influence FF have been of interest to various researchers over the years. Any factor that may affect the contribution of maternal or placental circulating cell-free DNA may affect FF. The effect may be either an increase in maternal circulating cell-free DNA concentrations, or a decrease in placental circulating cell-free DNA concentrations. Some biological factors are known to affect FF, while others are still under investigation. In addition to biological factors, experimental factors and FF calculation methods may also affect FF.

### Maternal Characteristics

FF is affected by multiple maternal factors (Table 1). The best-known factors affecting FF are maternal weight and gestational age. Many studies have found a negative correlation between FF and maternal body weight or body mass index (BMI). The FF of male fetuses (26, 28), female fetuses (26), and fetuses regardless of gender (24, 27, 29, 46) were negatively correlated with maternal body weight and BMI. After adjustment according to gestational age and maternal characteristics, BMI was found as a significant predictor of FF (47). Increased maternal body weight or BMI may increase maternal-derived circulating cell-free DNA concentrations, possibly owing to inflammation and necrosis of adipocytes (48), with or without a decrease in placenta-derived cffDNA concentrations (49). This may explain why the FF decreases with increased maternal weight or BMI. In the analysis of factors affecting FF, many researchers first corrected the FF using gestational age and BMI and then conducted multiple regression analyses. In this manner, the association bias caused by gestational age and BMI could be eliminated.

**Table 1:** Maternal factors that affect fetal fraction of circulating DNA.

**Positive correlation**	**Statistical values**	**Negative correlation**	**Statistical values**	**No correlation**
PAPP-A	0.1493 (0.0921-0.2064), <0.001^†^; Increased by about 1% per 0.5 MoM increase in PAPP-A (24); 0.133 (0.119-0.146), <0.0001^†^ (25)	Maternal body weight	−0.0093 (−0.0114 to −0.0071), <0.001^†^; Decreased by about 1% per 10 kg (24); *P* <0.001 (26); *P* <0.001 (27)	Maternal age (26)
Free β-hCG	0.0706 (0.0434-0.0978), <0.001^†^; Increased by about 0.4% per 0.5 MoM increase in β-hCG (24); 0.140 (0.128-0.152), <0.0001 (25)^†^	BMI	−0.541 (−0.697 to −0.385), <0.0001^†^ (28); −0.0022, <0.0001, 0.1241^‡^ (29); −0.295 (−0.329 to −0.26), <0.001^†^ (30)	Assisted reproductive pregnancy (31)
		Maternal age	−0.202 (−0.316 to −0.089), 0.0005^†^ (28); −0.081 (−0.103 to −0.059), <0.001^†^ (30); *P* <0.05 (32)	Serological screening risk (33, 34)
		Racial origin	African American (*P* = 0.007) (35); Asian women (*P* = 0.03) (36); South Asian, −0.019 (−0.032 to −0.005), 0.008^†^ (25)	Maternal smoking (37)
		Assisted reproductive pregnancy	−0.033 (−0.050 to −0.016), <0.001^†^ (38)	Low molecular weight heparin (39)
		Low molecular weight heparin	37.5, 11.19–125.87, <0.0001^§^ (40)	Pre-existing diabetes (41)
		Drug use	−0.6 (−1.2 to 0.1), 0.02^†^ (42)	Hyperthyroidism (41)
		Physical activity	*P* <0.01, a decrease varying from 1–17 percentage points (43)	Gestational diabetes (44)
		Maternal diseases	−4.1 (−5.7 to −2.5), <0.05^†^ (45)	HBsAg (30, 41)
		Pre-existing hypertension	RMoM of 0.85 (*P* = 0.02) (41)	

*PAPP-A, serum pregnancy-associated plasma protein; Free β-hCG, free β-subunit of human chorionic gonadotropin; BMI, body mass index; HBsAg, maternal carriers of the hepatitis B virus surface antigen; RMoM, the ratio between mean adjust multiples of the median value and theoretical “one”; aOR, the adjusted odds ratio adjusting for BMI, hypertension, anticoagulation use, and gestational age at circulating cell-free DNA blood draw*.

† *Regression coefficient (95% confidence interval), P;*

‡ *Regression coefficient, P; Intercept:*

§ *aOR, (95% confidence interval), P*. *Significant at P < 0.05*.

There is some controversy as to whether maternal age affects FF. FF was found to be negatively correlated with maternal age in many studies (28, 30, 32). Moreover, after adjusting FF according to gestational age and maternal characteristics, maternal age was found to be a significant predictor of FF (47). However, no correlation was found between FF and maternal age in other studies (26). Further research is needed on the relationship between FF and maternal age.

Another intensively investigated feature of pregnancy is the racial origin. One study conducted in the United States found that pregnant women with a lower FF were more likely to be African American (35). Furthermore, pregnant South Asian women generally had a lower FF than pregnant Caucasian women (25, 36). South Asian ethnicity was also a significant predictor of FF (47). These give us a hint that race also needs to be included in the discussion of the factors affecting FF in a multiracial society.

The relationship between FF in assisted reproductive pregnancy and that in natural conception is under continuous investigation. The concentrations of total circulating cell-free DNA and cffDNA and FF in assisted reproductive pregnancy were considered no different from those in the natural conception (31). However, singleton *in vitro* fertilization (IVF) fetuses were found to have a lower FF than naturally conceived fetuses in another study, and IVF was an independent predictor of a low FF and independently associated with test failure (38). Furthermore, the FF of assisted reproductive technology-treated women with the transfer of fresh embryos (mean gestational age is 89.7 days) is lower than that of frozen embryos (mean gestational age is 90.9 days) (29). This may be attributed to the relatively young gestational age of fresh embryos.

Some studies have found that low-molecular-weight heparin (LMWH) or enoxaparin use was associated with detection failure owing to a low FF (40). Treatment with LMWH may lead to apoptosis and thus decrease FF (40). Heparin has been shown to reduce trophoblast cell apoptosis and increase trophoblast cell survival by reducing new cytokeratin epitopes and nucleosome DNA formation (50), as well as E-cadherin expression (51) and other mechanisms. However, *in vitro* experiments showed that instead of heparin, autoimmune diseases in pregnant women were independent predictors of test failure (39). The exact mechanism of the interaction between LWMH and NIPT failures remains to be elucidated. New studies linking the FF to drug use in pregnant women have also become available. The FF was significantly lower in the group taking two or more drugs than in the group not taking any drugs. The group taking metformin had a 1.8% decrease in the FF (42). In addition, the FF present in blood samples taken immediately after completing a cycling exercise was significantly lower than that taken before cycling, with a decreased range of 1–17% (43). This may be attributed to the fact that the mother's circulating cell-free DNA concentration increased owing to exercise, while the cffDNA concentration remained unchanged, leading to a decreased FF. More papers containing enough samples are needed to support this conclusion. Maternal diseases or severe immune maternal disorders, such as systemic lupus erythematosus (52), B12 deficiency (53), severe thrombocytopenia and neutropenia (54), significant *HBB*-related hemoglobinopathies (45), and pre-existing hypertension (41) may also be associated with elevated maternal circulating cell-free DNA concentration or repeated detection failure owing to a low FF, while the FF improved after the disease was treated or suppressed.

Serological markers were also considered to be related to FF. Serum pregnancy-associated plasma protein (PAPP-A) and free β-subunit of human chorionic gonadotropin (free β-hCG) levels were positively correlated with FF (24, 25). The PAPP-A and free β-hCG levels in the test failure cohort were significantly lower than those in the success cohort. Moreover, the free β-hCG level was lower in the group with a low FF as the cause of test failure than in the group with other causes (55). However, there was no difference found in FF among high-risk, critical risk, and low-risk groups in serological screening (33, 34). This may reflect poor placental function or reduced placental volume. PAPP-A and free β-hCG are placenta-derived proteins that circulate in maternal blood (56). These placenta-derived proteins and cffDNA may be influenced by common factors, such as placental trophoblast cell mass (57) and the contact surface area between maternal blood and the placenta (56). This may be the reason why the PAPP-A and free β-hCG levels are associated with FF.

As has been noted, FF may be affected by many maternal factors, including BMI, maternal diseases or inflammatory states, race, and drug use. When high maternal BMI and maternal diseases or inflammatory states are present, it is necessary to be aware that FF may be low. The relationship between maternal age and assisted reproductive pregnancy and FF is unclear. In multiracial societies it is important to consider that FF varies between races. It is recommended to avoid the use of heparin anticoagulant blood for NIPS, and to avoid maternal use of heparin, multiple drugs and intense exercise before blood collection. The relationship between maternal factors and FF and the influencing mechanism needs further investigation. In general, maternal-induced FF changes are primarily attributed to an increase in maternal-derived circulating cell-free DNA concentrations or accompanied by a decrease in placental DNA concentrations.

### Fetal-Placental Characteristics

Fetal factors can also affect FF (Table 2). Gestational age at the time of blood collection is a key factor affecting this parameter. Various studies demonstrated that FF increased with gestational age, with a positive correlation between them (28, 30, 32, 58, 72). Gestational age was also found to be a significant predictor of FF (47). FF increased with gestational age throughout pregnancy. However, Hestand et al. found no correlation between FF and gestational age of up to 21 weeks (26). This may be because the increase in the FF is slow, and the increase rate is not constant. From 10 to 12.5 weeks of gestation, FF increased by 0.44% per week (46). Meanwhile, between 12.5 and 20 weeks of gestation, it increased at a rate of 0.083% per week. After approximately 20 weeks, FF increased steadily at a rate of 0.821%. The increase rate in the first trimester was lower than that in the second trimester. In addition, it is possible that although FF increased slightly with gestational weeks, it also decreased temporarily owing to the increase in maternal body weight (73). The fetal crown-rump length, an important parameter for calculating gestational age in the first trimester, as well as FF, also increases (7, 25, 34).

**Table 2:** Fetal-Placental factors that affect the fetal fraction of circulating DNA.

**Positive correlation**	**Statistical values**	**Negative correlation**	**Statistical values**	**No correlation**
Gestational age	0.959 (0.735 to 1.183), <0 .0001^†^ (28); 0.165 (0.127 to 0.204), <0.001^†^ (30); *P* <0.05 (32); Fetal fraction_ twin pregnancies (gestational age) = 0.646*gestational age + 4.360, R = 0.52^‡^ (58)	Trisomy 18	*P* = 0.04 (27); Ratio ^§^0.71, *P* <0.001 (12)	Gestational age (26)
Fetal crown-rump length	0.005 (0.002 to 0.009), 0.001^†^ (7); 0.001 (4.7E-04 to 0.001), <0.0001^†^ (25); Pearson correlation R^2^ = 0.023, *P* = 0.037 (34)	Trisomy 13	*P* = 0.004 (27)	Nuchal translucency thickness (15, 33, 37)
Female fetus	About 1% higher in pregnancies with female fetuses (24)	Twin pregnancies	−4.575 (−7.257 to −1.894), 0.0008^†^ (28); *P* <0.001 (59); *P* <0.0001 (60)	Fetal trisomy (61)
Trisomy 21	Ratio ^§^1.17, *P* <0.001 (12)	Hypertensive disorders of pregnancy/pregnancy induced hypertension	10.9 vs.12.4, *P* <0.0001 (62); Risk ratios = 1.6 [95%CI: 1.003-2.6] (63); *P* = 0.001 (35)	Low birth weight (35)
Twin pregnancies	Pearson correlation coefficient = 0.66, *P* <0.0001 (64); *P* = 0.0097 (65); *P* = 0.001 (66)	Preeclampsia	Risk ratios = 3.3 [95%CI: 1.2-8.9] (63); Adjusted OR = 2.06, 95% CI: 1.07-3.98 (67); OR = 0.59, 95%CI: 0.35-0.99, *P* = 0.048; and OR = 0.27, 95%CI: 0.08 to 0.96, *P* = 0.044) (62)	Placental abruption (35)
Preterm birth	Adjusted OR 4.59, 95% CI 1.39-15.2; adjusted OR 22.0, 95% CI 5.02-96.9 (16)	Fetal growth restriction	OR = 0.87, 95%CI: 0.79-0.96, *P* = 0.006 (68) *P* <0.001 (69)	Placenta accrete and placenta previa (70)
		Preterm birth	*P* = 0.002 (35); <34 weeks' gestation: adjusted OR = 3.09, 95% CI: 1.21-7.92 (67)	Intrahepatic cholestasis of pregnancy (67)
		Low birth weight	Adjusted OR of 2.32 (95% CI 1.15-4.67) for birth weight ≤ 10th percentile (*P* = 0.02) and aOR of 3.73 (95%CI 1.40-9.03) for birth weight ≤ 5th percentile (*P* = 0.004) (71); <2,500 g: adjusted OR = 2.50, 95%CI: 1.01-6.17 (67)	Gestational diabetes mellitus (44, 67)
		Placental compromise	Risk ratios = 1.6 and 95% CI: 1.1-2.2 (63)	

*95% CI, 95% confidence interval*.

† *Regression coefficient (95% confidence interval), P;*

‡ *The corresponding regression equations;*

§ *Ratio, median fetal fraction for subgroup/median fetal fraction for euploid pregnancies*. *^¶^ OR, odds ratio*. *Significant at P < 0.05*.

Research on FF in cases of multiple pregnancies remains limited (74). A large prospective, multicenter study demonstrated a FF range of 3–36% in twin pregnancies, with a mean FF of 12.2% (75). The optimal minimum FF that is valid for traditional NIPS in twin pregnancies should be 8%, although it is not certain that a sufficient FF is released from each fetus (76). The FF per twin was lower in some studies (59, 60) and higher in others (66), and the difference in FF contribution of each fetus may reach up to two-fold (77, 78). The existence of dichorionic twins was one of the influencing factors for the overall risk of test failure (9). The existence of dizygotic (DZ) twins was moderately correlated with FF. Moreover, the total FF of DZ twins and monozygotic twins was 35 and 26% higher than that of singleton fetuses, respectively (64, 65). The FF contribution per fetus in DZ twins was 32% less on average than that in singletons. Therefore, for twin pregnancies, establishing the zygosity of twins and determining FF in each fetus of DZ twins are important to achieve the optimal value of NIPS for aneuploidy screening.

In a case of a miscarriage of one twin, the DNA of the aborted fetus affected the FF. Vanishing twin is one of the reasons for testing failure. And the gestational age of blood sampling may be different between the IVF and natural conception for vanishing twin pregnancies (79). Some false-negative aneuploid cases detected through NIPS were attributed to a vanishing twin (80). Some testing platforms calculate the proportion of Y-chromosome reads to evaluate the FF. When the Y chromosome is at a static threshold, the Y-chromosome method will be used to calculate the FF. If the male fetus fails to live, and the living fetus is female, Y-chromosome contamination will gradually decrease as the pregnancy progresses, and the total FF will increase. Therefore, blood sampling after 14 weeks of gestation for NIPS may reduce the impact of a vanishing twin (81).

Pregnant women with female fetuses who undergo NIPS seem to have a higher FF in some studies (24). There was a marginal correlation (*p* = 0.067) between fetal trisomy and a low FF. However, there was no significant increase in the incidence of trisomy in the FF group with low FF detection failure (61). Conversely, FF was higher in the trisomy 21 group and lower in the trisomy 18 and trisomy 13 groups than in the euploid group, which may complicate the efficacy of NIPS in detecting trisomy 18 and trisomy 13 (12, 27).

A low FF was also found to be associated with an increased risk of placenta-related disorders and adverse perinatal outcomes. Pregnant women with an increased risk of hypertensive disorders of pregnancy and preeclampsia and those with preeclampsia with severe features had lower FF in early pregnancy (62), which was similar to the results of another research (63). Moreover, FF below the 10th percentile was associated with an increased risk of preeclampsia and early preterm birth <34 weeks (67), and FF greater than or equal to the 95th percentile was also associated with an increased risk of preterm delivery (16). Furthermore, FF below the 5th percentile was associated with an increased risk of low birth weight (67, 71). In addition, intrauterine growth restriction under 5th percentile was correlated with low FF (OR = 0.87, IC 95% 0.79-0.96, *P* = 0.006) (68), and the FF of pregnant women with early-onset growth restriction (2.00 ± 2.23%) was significantly lower than the expected FF (18.97 ± 10.17%) (69). This is consistent with the typical placental disorders of early fetal growth restriction. However, other complications, including placental abruption (35), placenta accrete and placenta previa (70), intrahepatic cholestasis of pregnancy (67), and gestational diabetes mellitus (44, 67), were not associated with FF.

In sum, FF may be affected by gestational age, fetal crown-rump length, fetal sex, fetal karyotype, and twin pregnancies, and FF is associated with an increased risk of placenta-related disorders and adverse perinatal outcomes. FF is positively correlated with gestational age, so NIPS should be performed at the appropriate gestational age. The position statement issued by the ACMG in 2016 indicated that NIPS can be used to screen fetuses for trisomy 21, trisomy 18, and trisomy 13 at a gestational age of 9-10 weeks, or more (22). For twin pregnancies, it is important to establish the zygosity of the twins and determine the FF of each fetus in DZ twins. Meanwhile, blood sampling after 14 weeks of gestation may reduce the effect of a vanishing twin (81). Because of these factors that may affect FF, clinicians should obtain as much detail as possible regarding the characteristics of the pregnant woman and fetus when considering NIPS. In this manner, comprehensive judgment can be made to ensure a sufficient FF and consequently an accurate NIPS screening for aneuploidy.

### Experimental Factors

Some pre-analytical aspects need to be considered. The serum FF was lower than the plasma FF, so plasma DNA is recommended for NIPS (82). Owing to maternal and fetal DNA fluctuations, FF in maternal plasma showed a downward trend after 2 years of storage at −25°C, decreasing after 3 years. However, the FF of the extracted DNA stored at −25°C for 18 months did not change, and the FF of the extracted DNA stored for 3 years increased by 27% compared with that detected immediately (83). After freezing, the plasma GC content in samples from pregnant women increased, and the FF, unit reads/total reads ratio, and Z score of trisomy 21 in male fetuses decreased, resulting in decreased detection accuracy (84). Some scholars have also studied changes in the FF caused by the use of different blood collection tubes. The proportion of cffDNA decreased steadily with the increase in storage time in conventional K3EDTA tubes, with a significant decrease of 48.5 and 65.7% on day 2 and day 3, respectively (85). A significant decrease of 80% was observed on day 4. An increase in the concentration of total circulating cell-free DNA in the plasma stored in circulating cell-free DNA BCT tubes at 4°C also resulted in a decrease in FF (86, 87). The FF in samples stored in STRECK BCT tubes may be higher than that in samples stored in EDTA tubes (88). STRECK BCT tubes may be the best blood collection tubes. Therefore, the impact of sample storage on FF before and during sample testing should be carefully considered, especially re-inspection of the frozen samples. Excessive transport or sample storage temperatures may rupture the mother's white blood cells, releasing more maternal cfDNA and resulting in a decrease in FF.

The experimental process of NIPS includes DNA extraction, library construction, and sequencing, and some test data in the experimental process may also affect FF (Table 3). FF was negatively correlated with the circulating cell-free DNA concentration, library concentration, and circulating cell-free DNA fragment size and positively correlated with uniquely mapped reads (28, 90). Therefore, when the circulating cell-free DNA and library concentrations are too high, the amount of fetal-derived cffDNA may be reduced, leading to a decrease in FF and thus affecting detection. The Z-value may also be positively correlated with FF (91, 92). Operator variation in performing the experiments during the testing may also affect FF, but it is not easy to quantify and standardize it. It may be considered that inter-and intra-operator variation in performing experiments during the testing is reflected in the data of the experimental process, such as circulating cell-free DNA concentration, library concentration, and uniquely mapped reads.

**Table 3:** Experimental factors that are associated with fetal fraction of circulating DNA.

**Positive correlation**	**Statistical values**	**Negative correlation**	**Statistical values**	**No correlation**
Uniquely mapped reads	2.292 (1.462 to 3.122), < .0001^†^ (28)	Circulating cell-free DNA fragment size	−0.695 (−0.78 to −0.61), <0.0001^†^ (28)	Hemolysis (89)
		Circulating cell-free DNA concentration	−1.05 (−2.45 to 0.34), <0.0001^†^^§^(90)	
		Library concentration	−4.86 (−6.48 to −3.23), <0.0001^†^^¶^ (90)	
Z-value	β = 0.77, SE = 0.04, *P* < 1*10-16 (91) rT21^‡^ = 0.905, *P*T21 = 0.00; rT18^‡^ = 0.887, *P*T18 = 0.00; rT13^‡^ = 0.858, *P*T13 = 0.01 (92)			

† *Regression coefficient (95% confidence interval), P;*

‡ *rT21: Pearson correlation coefficient between FF and the z-score of chromosome 21;*

§ *This reference presents a hierarchical regression analysis of the relationship between circulating cell-free DNA concentration and FF. Only the results of the group with the largest sample size (Circulating cell-free DNA Concentration <0.121 ng/ul) was shown here;*

¶ *Only the results of the group with the largest sample size (Library concentration > 10.701 ng/ul) was shown here*. *Significant at P < 0.05*.

To sum up, FF may be associated with circulating cell-free DNA fragment size, circulating cell-free DNA concentration, library concentration, uniquely mapped reads, and Z value. Therefore, laboratory staff should comprehensively analyze the data in the experimental process when examining the results.

### Methods for Calculation FF

Measurement of FF is essential for quality control. The approaches currently used for evaluating FF are summarized in Table 4. Among them, FF calculation methods based on the Y chromosome are the most common. They are also currently recognized as the most likely gold standard calculation method. However, these methods are limited to the calculation of FF in male fetuses only. Many studies have compared different FF calculation methods, showing significant differences in the FF among them.

**Table 4:** Approaches for evaluating fetal fraction (93, 94).

**Approaches**	**Algorithm name**	**References**	**Advantages**	**Disadvantages**
**Sex chromosome- based**				
Y Chromosome based			Easy and precise	For male fetuses only, not female fetuses
	Y Chromosome based/FFY	(3, 4, 34, 92)		
	DEFRAG	(26, 95, 96)		
	CSMART	(73)		
X-chromosome and Y Chromosome based	PREFACE	(97)	Training with a limited amount of retrospective data	NA
	BAYINDIR	(95, 98)		
**SNP-based**			Precise	Extra parental SNP information may not be readily available
Polymorphic loci quantified with microarray or sequencing	DANSR	(99–101)		
SNP loci		(102, 103)		
Insertion/deletion polymorphisms		(104)		
	SNPFF	(105, 106)		Underestimated FF less than 7%
**Sequence read count**	SeqFF	(26, 80, 95, 107)	Only sequencing of maternal circulating cell-free DNA; Applicable to regular noninvasive prenatal screening; sequencing is only required on a single end	Accuracy is not good at low FF; training with a large amount of retrospective data
**Differential methylation**		(108–110)	Precise	Further cost is added by whole-genome bisulfite sequencing; affected by methylation-sensitive restriction enzyme digestion and bisulfite transformation
**Fragment size based**		(111, 112)	Only sequencing of maternal circulating cell-free DNA; Applicable to regular noninvasive prenatal screening	Further cost is added by paired-end next-generation sequencing; moderate accuracy
**Nucleosome profile**	SANEFALCON	(26, 95, 96, 113)	Only sequencing of maternal circulating cell-free DNA	Lower accuracy; training with high-depth sequencing data
**Others**				
Maternal plasma DNA sequencing data with parental genotypes		(114, 115)	Direct and precise	Hindered by the requirement of parental genotypes
MPS deduction: sequence counts	FetalQuant	(116)	Targeting only the maternal circulating cell-free DNA; Precise	The sequencing depth is required to be as high as ~120 ×
Shallow-depth sequencing of maternal plasma DNA coupled with maternal genotypes	FetalQuant^SD^	(117)	Only shallow depth sequencing of maternal circulating cell-free DNA; Precise	The maternal genotype is required; Parameters of the model need to be reset according to sequencing and genotyping platform
Shallow-coverage sequencing of maternal plasma DNA	FF-QuantSC	(118)		

One study compared between DEFRAG (95) [including DEFRAG_W (entire Y-chromosome method) and DEFRAG_S (a subset Y-chromosome method)], SANEFALCON (113), and SeqFF (107) (including ENET (119) and WRSC (120) scores) tests for FF and discovered that only DEFRAG_W could accurately analyze fetal DNA distribution (26). Moreover, DEFRAG_S was more likely to report no or a high FF. However, SeqFF, ENET, and WRSC performed better in samples with a high FF. In addition, SANEFALCON had high false positives rates for FF. The SeqFF method cannot be limited to the evaluation of FF in male or female fetuses but may underestimate this parameter in high and low limits (105). Therefore, Hestand et al. concluded that DEFRAG_W was the best-tested method for calculating FF in male fetuses and SeqFF in female fetuses, although the latter still needs improvement (26). Moreover, the FF range in the SANEFALCON method was narrower than that in the DEFRAG method, and the assay-based method involving methylated RASSF1A promoters (96, 108). However, the DEFRAG assay can only detect FF in male fetuses, while the RASSF1A assay can detect the FF in both male and female fetuses but requires additional PCR steps.

Compared with the most reliable method FFY (4, 34), SNPFF underestimated FF by 7% (105), which was similar to the results of another study in which SNPFF underestimated FF by 10% (106). Moreover, Song et al. found that SNPFF underestimated the full range of FF (73). The SeqFFY method is equivalent to the FFY method in the case of a male fetus, and the FFY method corrects the SeqFF method in the case of a female fetus. In addition, the SNP-based FF calculation method is more costly. The sequence read count-based FF assessment methods (such as SeqFF) are based on the theory that cffDNA is more likely to come from regions with increased euchromatin DNA structure. Reads aligned within certain autosomal regions are counted and FF is calculated by weighting (107). The SNP-based FF evaluation method is to perform high-coverage targeted sequencing for highly polymorphic SNPs and evaluate FF from the SNP locations of maternal homozygous genes and fetal heterozygous genes (105). That's the difference between the two approaches. Moreover, FF calculated by fragment size-based method is highly consistent with that calculated by FFY (111). The fragment size-based method is based on the fact that the length of DNA fragments derived from the placenta is smaller than that of maternal origin. Both maternal and fetal cffDNA fragment size distributions show a series of peaks, including the main peak of 166 bp and a small peak of 143 bp, as well as peaks of less than 143 bp at intervals of 10 bp. The biggest difference between fetal cffDNA and maternal cfDNA is the decrease in the ratio of fragments of 166bp and the increase in the ratio of fragments less than 150bp (114). In addition, the consistency of the Y-chromosomal-based FF assessment methods is higher than that of methods that can also be used to measure the FF in female fetuses. The consistency was higher when only specific regions in the Y-chromosome were considered, including that of the DEFRAGb and BAYINDIRb (98) methods (95). The DEFRAGb method effectively identified a low FF. The two methods available for male and female fetuses were not as accurate as of the Y-chromosome method, and SANEFALCON was less effective than SeqFF. However, SANEFALCON also performed best in measuring a low FF, even better than did DEFRAGb. Therefore, the use of multiple FF calculation methods is recommended. For example, the SANEFALCON method was used to exclude samples with a low FF; thereafter, the DEFRAGb method and the SeqFF method were used to measure FF in male and female fetuses, respectively (95).

Re-evaluations using the FF-QuantSC method and analysis of distribution patterns of Y-chromosome reads may lead to more accurate results for samples with a repeatedly low FF, especially for derived chromosomes containing part of the Y-chromosome (121). In this manner, rare cases of sex reversal caused by *SRY* translocation could avoid being misjudged as detection failures by Y-chromosome-based FF calculation methods.

Each FF evaluation method has its advantages and disadvantages; however, it is difficult to determine which method performs the best or the worst. No method is perfectly efficient, and no method is inefficient. The FF calculation methods used by different testing platforms may differ, and the FF calculated by different methods is not currently directly comparable. Some methods also develop variants as experiments evolve. Moreover, different bioinformatics algorithms may affect the FF calculation methods (93). Therefore, it is important to be specifically based on the different methods that different laboratories use. It is not sufficient to use a single FF evaluation method. Instead, it is best to use a combination of multiple methods for FF evaluation: Instead of simply using FF thresholds as a quality control criterion, focusing on improving FF detection methods used by the testing platform takes precedence. There is also an urgent need for standardization of FF test methods and reporting (122, 123).

## Management of Pregnant Women With Failed Results Owing to a Low FF

It is important to address the issue of a low FF. Many studies have improved existing FF calculation methods. These improvements include enrichment of cffDNA, optimization of sequencing conditions, improvement of bioinformatics algorithms, and maximization of differences in maternal and fetal DNA fragment sizes (117, 124–128). Xue et al. used E-gel-based size selection NIPS to screen for and exclude shorter circulating cell-free DNA fragments from the total circulating cell-free DNA and then evaluated FF through Y-chromosome reads, which increased FF by 99–359% (128). After optimization, FF in a twin NIPS false-negative sample was increased by 23.1%. Abnormalities in two false-negative samples owing to confined placental mosaicism were also detected. Such improvements can improve the FF detection accuracy but could also increase the cost. Therefore, laboratories need to consider a trade-off. For clinical laboratories, there are a variety of calculation methods for FF, and many questions should be considered (Box 1). These are the clinical laboratory needs to be concerned. Since FF provides significant information, routine FF calculation is necessary when performing NIPS, although there is some debate regarding whether FF should be routinely reported. Every laboratory should establish a personalized test for FF determination and interpretation and establish a minimum detection threshold for obtaining valid NIPS results. Laboratory personnel can also predict in advance whether there will be a low FF according to the positive and negative correlation factors during the experiment so that the decrease of FF caused by the experimental factors could be minimized to some extent through the improvement of the experiment.

BOX 1 **Fetal fraction toolbox for genetic counselors and clinical laboratories providing NIPS:**

**Questions for genetic counselors to ask themselves**

What are the basic characteristics of the pregnant woman and fetus that need to be collected before conducting NIPS?

Are there maternal or fetal factors that may cause increased FF detected by NIPS?

Are there maternal or fetal factors that may cause decreased FF detected by NIPS?

How to manage pregnant women with failed results owing to a low FF?

**Questions for clinical laboratories providing NIPS to ask themselves**

Is FF routinely calculated and is a minimum detection limit for the FF calculation set?

What is the calculation method used and what are its advantages and disadvantages?

Is FF routinely reported in NIPS reports and is its significance indicated in clinical reports?

What is the detection failure rate owing to a low FF?

Is the cause of test failure reported?

What is the detection success rate of blood redrawn after test failure?

Are additional follow-up tests provided after NIPS fails?

**Figure 2:**
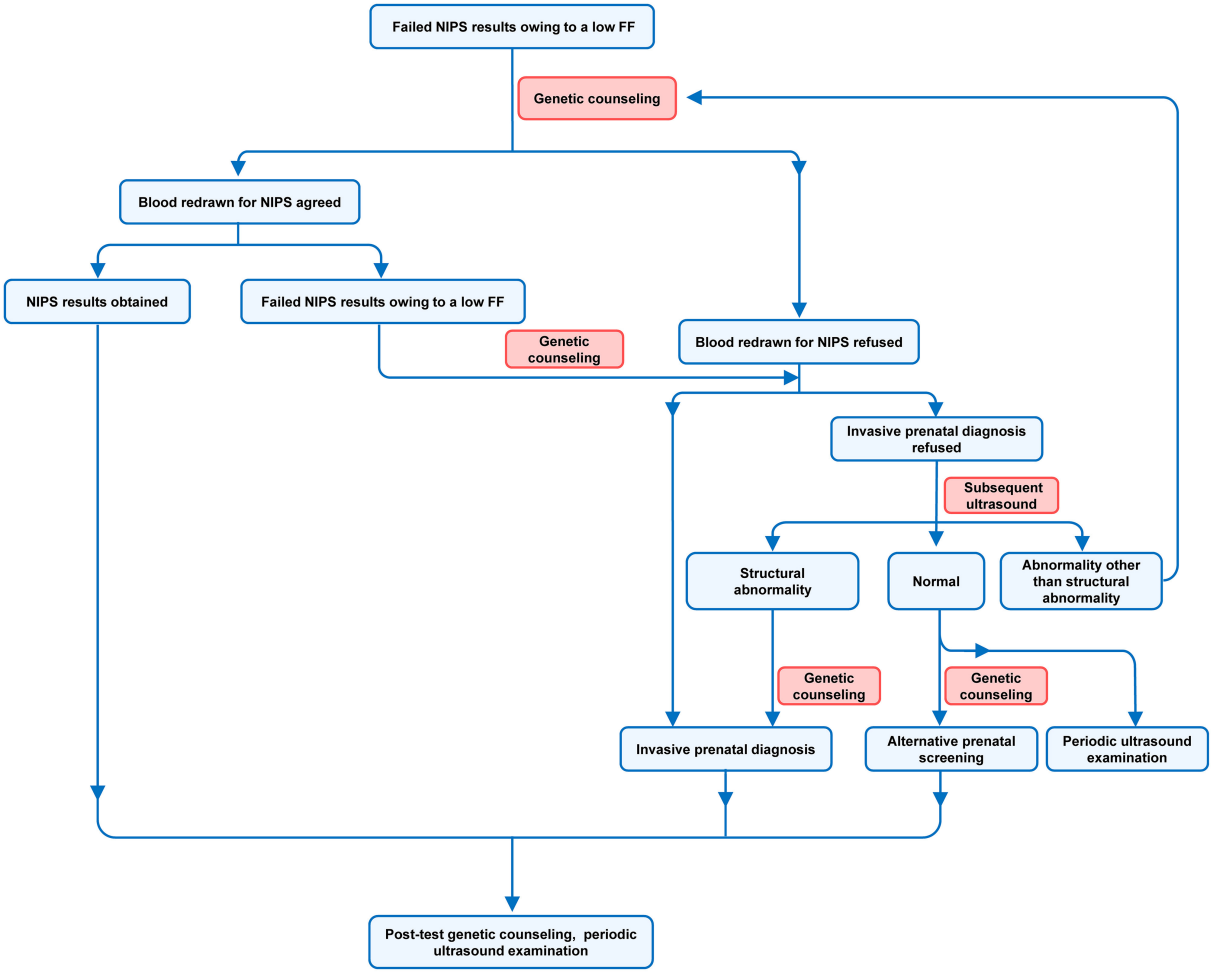
Management procedures of pregnant women with failed results owing to a low FF.

Genetic counselors are supposed to focus on what they need to know (Box 1). Various NIPS and FF evaluation methods are currently available, and genetic counselors do not need to know the specific methods. However, they are expected to be aware that the FF obtained through different methods and different laboratories cannot be directly compared, emphasizing the need for caution when interpreting NIPS reports. When collecting information on pregnant women, genetic counselors are supposed to make a basic judgment on whether a high or low FF will be obtained in NIPS and how to manage the result. When a pregnant woman receives a “no call” NIPS result, subsequent genetic counseling is recommended (Figure 2). Genetic counselors are expected to discuss the factors that are associated with fetal fraction of circulating DNA and also provide professional advice on the cause of test failure. The reason for a low FF may be the increase of factors negatively related to FF, such as BMI, or the decrease of factors positively related to FF, such as gestational age. Pregnant women ought to be advised that NIPS failure may be influenced by factors affecting FF or may indicate an increased risk of fetal aneuploidy. Pregnant women can decide to have their blood redrawn for NIPS and attempt to avoid factors that may affect FF. However, some factors affecting FF are unavoidable; thus, a 40–50% test failure rate from resampling remains feasible (94). The NIPS success rate from resampling was mainly determined by the FF of the initial sample and the pregnant woman's weight (102). Based on the FF of the initial sample, maternal weight, and interval between blood collections, a personalized assessment of the test success rate of resampling for each pregnant woman could be considered (129). This is attributed to the strong correlation between the FF of the same blood sample from the same individual person and the FF of different blood samples (46). Based on the resampling results, subsequent management strategies are then suggested by genetic counselors.

Invasive prenatal diagnosis remains an option if a pregnant woman is unwilling to have her blood redrawn for NIPS. If the pregnant woman opts against a redraw and an invasive prenatal diagnosis, the decision is based on the subsequent ultrasound results and the pregnant woman's judgment. If the ultrasound results are normal, alternative prenatal screening, such as combined first-trimester screening, may be an option (94). However, if the ultrasound results are abnormal, the pregnant woman may need to have her blood redrawn for NIPS or undergo an invasive prenatal diagnosis. If the ultrasound results present a structural abnormality, a direct invasive prenatal diagnosis is recommended. Periodic ultrasound examination is necessary regardless of whether blood is redrawn for NIPS. If the NIPS still calculates a low FF following the redraw, invasive prenatal diagnosis is recommended, and regular ultrasound should be maintained.

In samples with a significantly reduced FF, the most common abnormalities were trisomy 18, trisomy 13, and triploidy (12, 25). However, these abnormal fetuses will likely present abnormalities on subsequently targeted ultrasound, such as a heart defect or limb abnormality (130, 131). Therefore, it is not necessary to perform invasive prenatal diagnosis for every pregnancy whose NIPS test fails owing to a low FF. However, if this NIPS test failure is secondary to other high-risk outcomes, such as advanced age or serological screening, the recommendation for invasive prenatal diagnosis may be appropriate. In general, it is essential that the genetic counselors closely liaise with the clinical laboratories to manage pregnant women who received failed NIPS results. It is recommended that the clinical laboratories report the cause of test failure and that the genetic counselors conduct comprehensive genetic counseling for pregnant women.

Therefore, genetic counseling of pregnant women with failed results owing to a low FF requires the full involvement of the genetic counselors. After failed NIPS results due to low FF obtained, the genetic counselors are supposed to make recommendations based on the characteristics of each pregnant woman and the test results during pregnancy, then the pregnant woman could choose further tests based on the professional information provided by the genetic counselors and her demands.

## Conclusions

cffDNA is the cell-free fetal DNA present in maternal plasma and forms the basis of NIPS. FF is an important quality control link of NIPS. It is necessary to have a specific understanding of FF to maximize the value of NIPS. A high or low FF has different meanings, and a normal range of FF is very important for obtaining accurate NIPS results. Furthermore, FF is affected by many factors, including maternal characteristics, fetal-placental characteristics, experimental factors, and calculation methods. Both genetic counselors and laboratory staff should contribute to obtaining accurate NIPS results. When test failure of NIPS due to low FF is obtained, the genetic counselors and clinical laboratory are supposed to work together to manage the pregnant women. Going forward, a combined effort is needed to reduce NIPS failure owing to a low FF.

## Author Contributions

CD and SL had the idea for the article. CD performed the literature search and data analysis and drafted the work. SL critically revised the work. All authors contributed to the article and approved the submitted version.

## Funding

This work was supported by the National Key Research and Development Program of China (2018YFC1002203) and the Technology Research and Development Program of the Science and Technology Department of Sichuan Province, China (2021YFS0078).

## Conflict of Interest

The authors declare that the research was conducted in the absence of any commercial or financial relationships that could be construed as a potential conflict of interest.

## Publisher's Note

All claims expressed in this article are solely those of the authors and do not necessarily represent those of their affiliated organizations, or those of the publisher, the editors and the reviewers. Any product that may be evaluated in this article, or claim that may be made by its manufacturer, is not guaranteed or endorsed by the publisher.

## Abbreviations

NIPS: Noninvasive prenatal screening
FF: Fetal fraction
cffDNA: Cell-free fetal DNA
ACMG: American college of medical genetics and genomics
BMI: Body mass index
IVF: *in vitro* fertilization
LMWH: Low-molecular-weight heparin
PAPP-A: Pregnancy-associated plasma protein
free β-hCG: free β-subunit of human chorionic gonadotropin
HBsAg: Carriers of the hepatitis B virus surface antigen
95% CI: 95% confidence interval.
